# Accelerated endochondral growth in adolescents with idiopathic scoliosis: a preliminary histomorphometric study

**DOI:** 10.1186/1471-2474-15-429

**Published:** 2014-12-13

**Authors:** Xin Zheng, Weijun Wang, Bangping Qian, Shoufeng Wang, Zezhang Zhu, Bin Wang, Xu Sun, Yitao Ding, Yong Qiu

**Affiliations:** Department of Spine Surgery, The Affiliated Drum Tower Hospital of Nanjing University Medical School, Zhongshan Road 321, Nanjing, 210008 China

**Keywords:** Idiopathic scoliosis, Growth plate, Iliac cartilage, Endochondral ossification

## Abstract

**Background:**

Abnormal longitudinal growth has been identified in the early pubertal stage of idiopathic scoliosis (IS) and is thought to contribute to the development of scoliosis. This phenotype may be caused by abnormal endochondral ossification, but histological evidence is lacking. The aim of this study was to investigate whether there is abnormal endochondral ossification in IS patients at early stage of puberty by histomorphometric analysis of their iliac cartilage.

**Methods:**

Fifty-two patients with IS and 19 controls were recruited and grouped according to their Risser grade (Group A: Risser grade 0 with Oxford stage 2–3; Group B: Risser grade 2). Group A consisted of 20 IS patients (mean age: 12.3 years) and 9 controls (mean age: 12.0 years), while Group B included 32 IS patients (mean age: 13.8 years) and 10 controls (mean age: 13.7 years). Biopsies of the iliac cartilage were harvested intra-operatively and prepared using routine histological methods. Histomorphometric analysis was performed to quantify the thickness of the hypertrophic zone, the area and number of chondrocytes in the cell-nest, and the number of chondrocytes in the proliferative zone using Image-Pro Plus software.

**Results:**

In Group A, a significantly thicker hypertrophic zone and larger cell-nest area and number of cells within the cell-nest, and in the proliferative zone, were found in iliac cartilages from IS patients compared with those of controls (all *P* < 0.05). In group B however, there were no significant differences in histomorphometric parameters between IS patients and the controls.

**Conclusions:**

The differences in the histomorphometric results between IS patients and their controls for patients with Risser grade 0 and Oxford grades 2 & 3, but not in those with Risser grade 2, indicated a pattern of accelerated endochondral growth in IS at the early stage of puberty, but not at the late stage.

**Trial registration:**

Current Controlled Trials: ChiCTR-CCC-13003988. Registered 17 December 2013. http://www.chictr.org/usercenter/project/edit.aspx?proj=6233.

**Electronic supplementary material:**

The online version of this article (doi:10.1186/1471-2474-15-429) contains supplementary material, which is available to authorized users.

## Background

Idiopathic scoliosis (IS) is a three-dimensional spinal deformity that is characterized by significant curve progression during the growth spurt. Consensus has been reached that abnormal longitudinal growth plays an important role in the development and progression of IS [1–11]. The majority of anthropometric measurements [8, 12–14] show that patients with idiopathic scoliosis tend to be taller than their counterparts, although a few studies [15, 16] report comparable heights between IS patients and healthy controls. In addition to the faster growth of IS patients during puberty that was recorded in a longitudinal study [17], a relative anterior spinal overgrowth in girls with IS was also revealed by magnetic resonance imaging (MRI) [4, 18]. More importantly, a histomorphometric study of the vertebral endplates from IS patients confirmed a more active growth of the anterior column than of the posterior spinal column [11]. All these observations support the presence of abnormal systemic growth and the probability of abnormal regulation and modulation of skeletal growth in patients with IS.

In a cross-sectional study of anthropometric parameters in 598 adolescent girls with IS and 307 maturity-matched healthy girls, Cheung *et al.*[8] found that when the subjects were in the pre-pubertal stage (Tanner stage I), the girls with IS were shorter than their peers. However, during the pubertal growth spurt (Tanner stage II to IV), the girls with IS tended to be taller and have longer arm spans than the controls [8]. Goldberg *et al.*[19] also found that younger girls with IS were taller than their peers, but no differences in height were found later in adolescence. Hagglund *et al.*[5] have also noted a higher rate of growth during the two years before the onset of puberty in girls with IS when compared with controls.

Based on this previous work, we hypothesized that there is an accelerated endochondral growth in the early pubertal stage of IS. Although a common assumption, no anthropometric measurements have been reported in histologic studies to provide evidence for it. Skeletal growth in IS is well-accepted as a systemic abnormality [8, 10, 20]. Therefore, histomorphometric analysis of the endochondral activity of the iliac crest cartilage was used with the aim of determining whether the growth potential of patients with IS was higher than that of controls during the early stage of puberty.

## Methods

This study was approved by the Medical Ethics Committee of Nanjing Drum Tower Hospital. Written consent for participation in this study was obtained from all patients and their parents. Adolescents with IS indicated for posterior spine surgery with autogenous iliac crest bone grafting and non-IS patients indicated for iliac bone harvesting were recruited for this study. The diagnosis of IS was made by medical history, physical examination, and radiologic investigation. The inclusion criteria were: 1) age 10–16 years; 2) Risser grade 0 or 2 (due to the limited number of patients in our cohort, those with Risser grade 1 were not included); and 3) triradiate cartilage stage 2–3 (Oxford method) [21]. Patients with metabolic bone disease, growth disturbance (e.g. osteochondrodysplasia and congenital heart disease) were excluded, as well as patients with previous spine or pelvic surgery. Non-IS patients with whole spine posterior–anterior radiographs to exclude scoliotic deformity were assigned as controls.

All X-rays were taken on the same machine and evaluated by a single person for precise evaluation. For adolescents with IS, the curve magnitudes were measured using the Cobb method [22] on the standing posterior–anterior X-ray films, which was taken within one week before surgery, and the most severe curve was used to present curve severity. The curve types were identified according to the location of the major curve apex as single thoracic, single thoracolumbar/lumbar, double thoracic, double major (thoracic and lumbar/thoracolumbar), or a multiple curve pattern of three or more curves [23].

In both the IS and control groups, the clinical profiles including gender, chronological age, and the menarchal status and years since menarche (YSM) for girls, were recorded. The skeletal maturity of the subjects was assessed by Risser grades and the status of the triradiate cartilage. Risser’s grade was estimated using X-ray films taken one week before the operation by a single experienced spine surgeon according to the ossification grade of the iliac apophysis [24], and it was assessed by the American version of the Risser grading system [25] in this study. The status of triradiate cartilage ossification was assessed using the Oxford method [21]: stage 1 indicates a widely open triradiate cartilage, stage 2 is the first sign of osseous invasion of the triradiate cartilage itself, and stage 3 represents total ossification.

Because we sought to compare the growth activity of the growth plate chondrocytes of IS patients with controls, considerable remaining growth potential was required in the patients. According to our analysis of the growth parameters, the pubertal growth of the adolescents could be characterized by two phases: an acceleration of growth during the early stage, followed by a deceleration of growth in the late stage [26]. The two phases were identified using the ossification status of the iliac epiphyses (Risser sign). The pubertal growth is in the deceleration phase once there is ossification in the iliac epiphyses (Risser ≥1), which is why we recruited patients with Risser grades 0 and 2. The growth activity of IS patients and controls were compared at both early (Risser grade 0 with Oxford stage 2–3) and late (Risser grade 2) pubertal stages in the present histomorphometric study of the iliac cartilage.

### Sample harvesting and preparation

Each iliac crest apophysis specimen was taken 20 mm lateral to the posterosuperior iliac spine for autogenous grafting. A standard block of 5 mm length × 5 mm width cartilage with a thin layer of fibrous connective tissue and cancellous bone was removed for this study. These specimens were immediately fixed in 4% paraformaldehyde for 24 hours followed by decalcification in 0.5 M ethylenediamine tetraacetic acid for 2 weeks. After confirming the decalcification with X-ray film, the specimens were dehydrated in serial ethanol solutions and then embedded in paraffin. The embedded blocks were sectioned to 5 μm perpendicular to the calcification layer. The sections were stained with hematoxylin and eosin (HE) to visualize the different zones of the growth plate cartilage.

### Histomorphometric analysis

**Figure 1:**
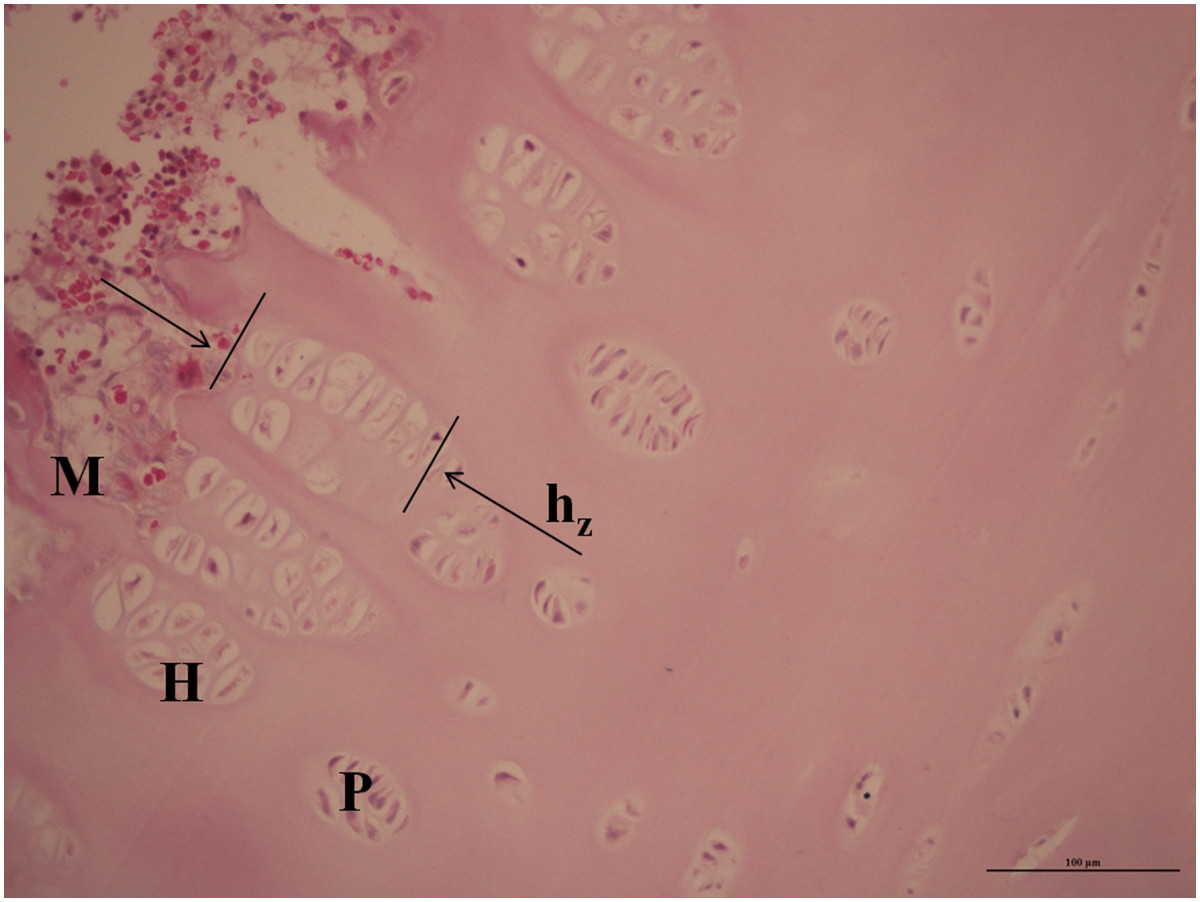
**The typical zonal structure of the growth plates.** The thickness of the hypertrophic zone (h_z_) was defined as the distance from the top edge of the most cephalic qualifying cell to the bottom edge of the most caudal one. Magnification: ×200; H: hypertrophic zone; M: mineralization zone; P: proliferative zone.

Three well-stained sections were selected for each subject and imaged by light microscopy. All sections were histomorphometrically analyzed by two separate pathologists independently, and the results from the two evaluations were averaged. The inter-observer reliability was accessed by calculating the intraclass correlation coefficient (ICCC) and the average ICCC values which represented interobserver variability was 0.900. The resting, proliferative, and hypertrophic zones of the growth plate were identified on the images according to the cellular morphology of the chondrocytes [27]. The hypertrophic zone was defined as the area in which the chondrocytes were larger and lighter-stained than those in the proliferative zone [27]. The thickness of the hypertrophic zone was defined as the distance along the longitudinal axis of the local growth plate boundaries in the direction of the cell columns from the top edge of the most cranial cell to the bottom edge of the most caudal cell. It was well-documented by several studies [16–18] that the rate of longitudinal bone growth is determined primarily by the activity of the chondrocytes in the proliferative and hypertrophic zones. Therefore, we quantified those two zones here. To quantify the differences between groups, the thickness of the hypertrophic zone and the area of the cell-nests within this zone were measured with Image-Pro Plus software (version 6.0, Media Cybernetics, Inc., USA) (Figure 1). The area and number of chondrocytes in each cell-nest and the number of chondrocytes in the proliferative zone were calculated. Only cells with clear boundaries were included.

Statistical analysis was performed using the SPSS (version 13.0; SPSS, Inc., Chicago, IL, USA) software package. Data were compared using Students’ *t*-tests if data normally distributed, *Chi-square* tests or non-parametric tests if data not normally distributed. For non-parametric tests, the Mann–Whitney U test was used and results were expressed as median (minimum, maximum). P-values less than 0.05 were considered statistically significant.

## Results

From July 2008 through July 2012, 52 adolescents with IS and 19 controls were recruited for this study. In patients with IS, the curve patterns were identified as single major thoracic in 29 patients, single thoracolumbar/lumbar in 11, double major thoracic in seven, and thoracic and lumbar/thoracolumbar in five. For the controls, nineteen cases without spinal deformity were enrolled, including osteoid osteoma of the lumbar spine in two patients, sacrum tumor in two, spine fracture in five, intraspinal neurilemmoma in one, lumbar spondylolisthesis in six and pelvis fracture in three. The clinical features of the IS patients and controls are shown in Table 1. The gender distributions between the IS patients and the controls was not significant in either group.

**Table 1:** **Clinical characteristics of the IS and control subjects**

	IS (n = 52)	Control (n = 19)	P value
Age	13.2 ± 1.3 (11.0–16.0)	12.6 ± 1.9 (10.7–16.0)	0.181
Gender			
Group A			0.158
Male	3	4	
Female (*postmenarchal*)	17 (6)	5 (1)	
Group B			0.135
Male	3	3	
Female (*postmenarchal*)	29 (16)	7 (3)	
Risser grade			0.499
0	20	9	
2	32	10	
Oxford stage (Risser = 0)			0.436
2	8	5	
3	12	4	
Cobb angle (°)	54.1 ± 14.6 (40–105)	-	

### Maturity assessment

In subjects with Risser grade 0 and Oxford stage 2–3, the mean chronological age of the IS group was 12.3 ± 0.8 years and of the control group was 12.0 ± 1.2 years. Six girls in the IS group and 2 in the control group had experienced menarche, with mean YSMs of 3.9 and 3.0 months, respectively. There was no significant difference in chronological age or YSM between the IS and control patients. There was also no significant difference in the Oxford grades of the triradiate cartilage between the IS and control patients (χ^2^ = 1.163, *P* = 0.281).

In subjects with Risser grade 2, the mean chronological age of the IS group was 13.8 ± 1.2 years and of the control group was 13.7 ± 1.8 years. Twenty-three girls in the IS group and 5 girls in the control group had experienced menarche, with mean YSMs of 5.7 and 5.2 months, respectively. The difference of the chronologic age and YSM was also not significant between IS patients and controls.

### Histomorphometric analysis of the growth plates

**Figure 2:**
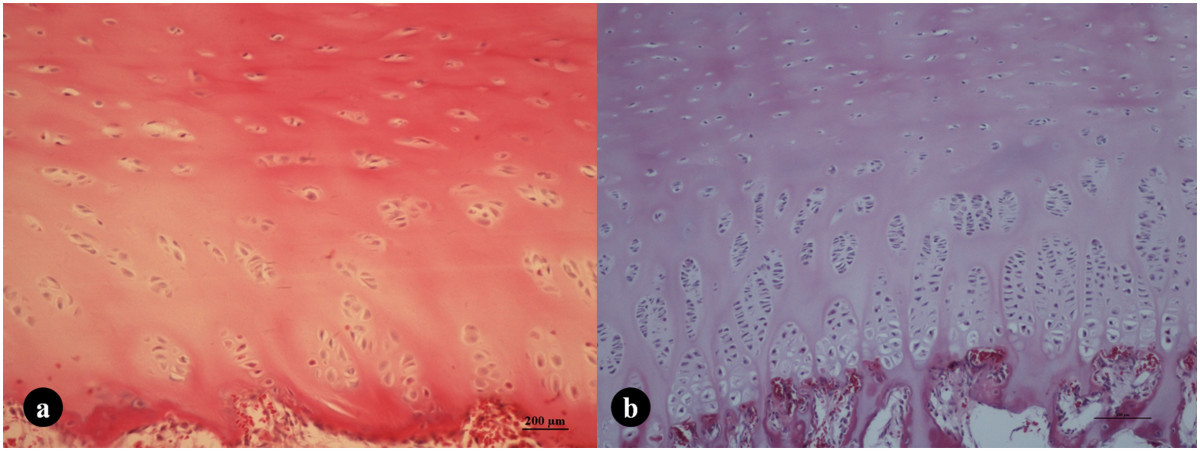
**The histological features of the iliac cartilage growth plates in patients with Risser grade 0. a)** A girl with L5 spondylolisthesis, (age: 12.3y, premenarche, Oxford stage 2). **b)** A girl with IS (age: 12.5y, premenarche, Oxford stage 2). The typical zonal structure of the growth plates was found in both subjects. In the hypertrophic zone, chondrocytes are organized in columns of cells that undergo differentiation, demonstrating remarkable growth activity in both subjects. A thicker hypertrophic zone, larger cell-nest area, and higher number of cells were observed in IS patients with Risser grade 0 **(b)** than in control patients with the same Risser grade **(a)**. HE, 200×.

The results of the quantitative histomorphometric analysis of all subjects are shown in Table 2. Non-parametric statistical tests were used because the data were not normally distributed. In subjects with Risser grade 0 and Oxford stage 2–3, the IS patients had significantly thicker hypertrophic zones (270.8(230.2, 307.4) μm vs. 222.6(169.3, 277.5) μm, *P* < 0.05), larger areas of the cell-nest (6566.8(3656.1, 10306.7) μm^2^ vs. 5433.2(2152.5, 6734.6) μm^2^, *P* < 0.01), and higher numbers of cells in the cell-nest (14.2(10.4, 16.6) vs. 10.1(9.5, 12.0), *P* < 0.001) than those of skeletally mature matched controls. The number of chondrocytes in the proliferative zone was also higher in IS patients than in controls (111.8(91.7, 140.1) vs. 86.6(71.4, 110.3), *P* < 0.05) (Table 2, Figure 2a, b).

**Table 2:** **Histomorphometric analysis of the iliac cartilage of IS and control subjects (expressed as median (minimum, maximum))**

	HZ thickness (μm)	Area of cell-nest in HZ (μm^2^)	Number of HC (/cell-nest)	Number of PC (/microscope)
IS (Risser = 0)	270.8(196.9, 318.0)	6566.8(3009.9, 10306.7)	14.2(8.0, 16.6)	111.8(79.0, 202.0)
Control (Risser = 0)	226.6(91.0, 328.4)*	5433.2(1850.7, 19223.0)*	10.1(5.3, 13.1)*	86.6(67.0, 131.0)*
IS (Risser = 2)	182.8(134.0, 310.0)	4721.1(2347.0, 13006.0)	9.0 (6.4, 14.8)	80.7(67.0, 110.0)
Control (Risser = 2)	165.5(138.0, 194.0)	4388.1(2581.8, 11544.0)	8.7 (6.5, 11.5)	78.6(62.0, 83.0)

HZ: hypertrophic zone; HC: chondrocytes in hypertrophic zone; PC: chondrocytes in the proliferative zone; IS: idiopathic scoliosis.

The Mann–Whitney U test were used to compare IS and control subjects, *:P < 0.05.

**Figure 3:**
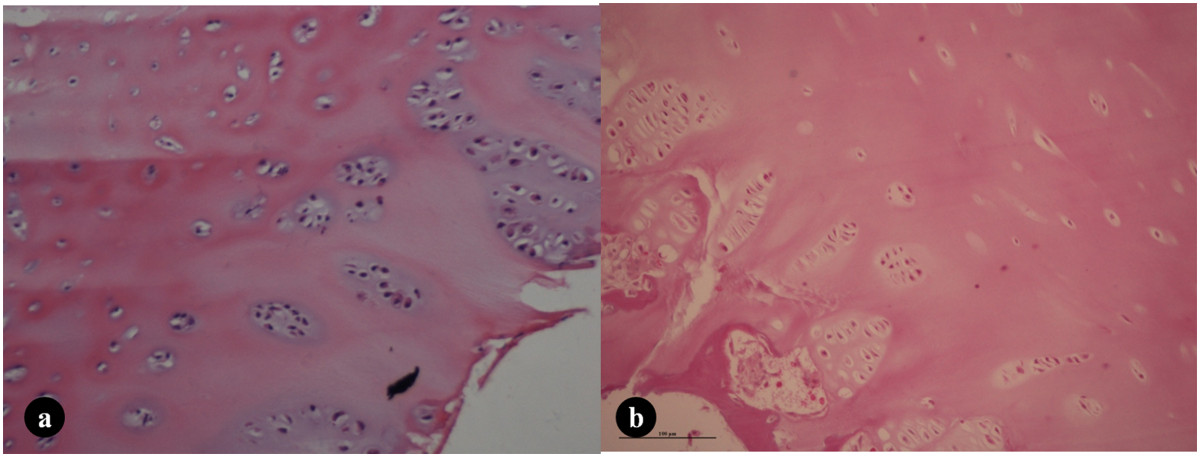
**The histological features of the iliac cartilage growth plates in patients with Risser grade 2. a)** A girl with sacrum tumor (age: 13.7y, 4 month postmenarche Risser 2). **b)** A girl with IS (age: 14.3y, 3 month postmenarche, Risser 2). A thinner hypertrophic zone, smaller area of cell-nest, fewer cells in the cell-nest in the hypertrophic zone, and fewer chondrocytes in the proliferative zone were found in both patients, indicating less growth activity. No significant differences were identified between the IS **(b)** and control **(a)** patients with Risser grade 2. HE, 200×.

In patients with Risser grade 2, the thickness of the hypertrophic zone, the area of the cell-nest within it, and the average number of cells in the cell-nest were not different between IS patients and the controls (all *P* > 0.05). In addition, the difference in the number of chondrocytes in the proliferative zone between IS and control patients was also not statistically significant (80.7(73.3, 87.0) vs. 78.6(70.6, 85.9), *P* = 0.644) (Table 2, Figure 3a, b).

In both the IS and control subjects, the growth plates of Risser grade 2 subjects showed significantly lower thickness, area of cell-nest, number of cells in the cell-nest in the hypertrophic zone, and number of chondrocytes in the proliferative zone compared with those of subjects with Risser grade 0 and Oxford stage 2–3. These results indicate that the growth activity of subjects with Risser grade 2 was lower than that in subjects with Risser grade 0 and Oxford stage 2–3 in both groups.

## Discussion

Although there are no generally accepted explanations for the etiopathogenesis of IS, one well-accepted concept is the presence of abnormal skeletal growth in patients with IS [10, 25]. Compared with controls, IS patients have been characterized as taller and leaner with longer arm spans [8, 12–14]. Focusing on the early and late phases of pubertal growth, several cross-sectional studies have reported significantly taller IS patients with longer arm spans at the early, but not in the late, pubertal growth stages when compared with age-matched healthy controls [9, 10, 20, 28, 29]. Consistent with these findings, a longitudinal study conducted by Goldberg *et al*. [19] demonstrated that younger girls with IS were taller than their peers, but the difference is lost by late adolescence. In addition, a significantly higher peak height velocity was found in girls with IS than controls (8.1 cm/year vs. 7.1 cm/year) [14], which implies a much faster growth during the early stage of pubertal growth.

To confirm that there is activated growth in IS patients during the early pubertal stage, IS patients and maturity-matched controls were recruited into the present study, and histomorphometric analysis of the iliac crest cartilage was performed. We used the iliac crest cartilage instead of the vertebral growth plate to represent the general growth activity here. One reason for this was our concerns regarding changes to the mechanical loading on the scoliotic spine [28]. Vertebral growth is partially governed by the Hueter-Volkmann law. The growth activity is regulated by the amount of compression: growth is retarded by increased compression, but accelerated by reduced compression [29]. Scoliotic deformities are thought to produce an asymmetrical stress distribution across growth plates and to cause asymmetrical growth in a “vicious cycle” [30]. Though the pelvis is “the most caudal vertebra”, and therefore potentially asymmetrically loaded [31], the mechanical loading of the iliac crest may be less affected by spinal deformity. Harvesting healthy vertebral growth plate from normal control patients is ethically problematic. Furthermore, abnormal longitudinal growth in IS patients is thought to be systemic problem that could also exist in other parts of the skeleton besides the spine. For these reasons, the iliac cartilage was selected for the histomorphometric study to reflect the general endochondral growth activity of both IS and control patients.

The pubertal diagram is characterized by a two-year ascending phase followed by a three-year descending phase [25]. An increased growth velocity associated with a significant height gain is found during the ascending phase, when there is no ossification in the iliac crest apophysis in patients with Risser grade 0. The growth velocity decreases during the descending phase as the ossification of the iliac crest apophysis begins. Hence, Risser 1 heralds the beginning of the descending slope of the pubertal growth velocity diagram. In the present study, the subjects with Risser grade 0 and Oxford stage 2–3 represent the early stage of puberty, the ascending phase of longitudinal growth, while subjects with Risser grade 2 correspond to the descending phase of the pubertal diagram [25]. Therefore, the iliac cartilage from IS patients and controls with Risser grades 0 (and Oxford stage 2–3) and 2 represent growth activity at the early and late stages of pubertal growth. To our knowledge, no data comparing the histological characteristics of growth plates between IS and control patients has been reported in the literature.

Using MRI, higher vertebral bodies and relatively longer total vertebral lengths, but similar total spinal cord length, have been demonstrated in girls with IS compared with maturity-matched controls [4, 6, 18]. In a comparative investigation by MRI of the morphometry of thoracic vertebrae in 83 girls with IS and 22 age- and gender-matched normal subjects, Guo *et al*. [4] found a relatively faster growth of anterior, and slower growth of posterior, elements of the thoracic vertebrae in the patients with IS compared with the normal controls. These findings imply that, compared with age-matched controls, the longitudinal growth of the vertebral bodies in patients with IS is disproportionately faster. Because the longitudinal growth of the vertebral column and peripheral bones during puberty mainly occurs by endochondral ossification of growth plates 30, the above findings suggest that there may be greater proliferative activity in IS patients compared with normal controls [4, 18].

Chondrocyte hypertrophy plays an important role in the longitudinal growth of the skeleton [32]. Increases in chondrocyte height are responsible for 44 to 59% of long-bone growth, with the remainder being due to matrix synthesis and chondrocyte proliferation [33, 34]. A positive linear relationship between the rate of longitudinal bone growth and the final volume of hypertrophic chondrocytes was demonstrated by Breur *et al*. [35]. Developed for analysis of growth plates, histomorphometry is commonly used for histologic quantification of the relative thicknesses of the zones of proliferating and hypertrophic chondrocytes [36]. By comparing the growth activity of the spinal growth plates from the anterior and posterior column of the spine in IS using histomorphometric analysis, Zhu *et al*. [11] found that the proliferative and hypertrophic chondrocytes in the anterior spinal column of IS patients were more active than those of the posterior column. However, the results of Zhu *et al*.’s study were based on a comparison between AIS and congenital scoliosis (CS) patients. Because the structural vertebral anomalies that caused the CS deformity may also affect growth plate development, it was inappropriate to consider the growth plates of the spine from CS patients as normal references. Moreover, as suggested in other reports in the literature, the abnormal growth in IS patients has been implicated as systemic. Not only were IS patients found to be taller than their controls, other anthropometric measurements such as arm span, radius length, and radius diameter were also different between girls with IS and control subjects [9, 37]. These previous studies by Guo *et al*. [4] and Zhu *et al*. [11] concentrated on the spine, which does not reflect the systemic abnormal growth in IS patients because of the asymmetric mechanical loading on the scoliotic spine.

As Schwender *et al*. [38] and Burwell *et al*. [39] reported, even without leg length discrepancies, some AIS patients had iliac obliquity, which may be explained by intrinsic changes in the pelvis. There may be a left-right asymmetry of growth activity at the pelvis because anatomic iliac wing asymmetries were found in marked scoliosis [39]. While in Burwell *et al*.’s study [37], upper arm length (UAL) asymmetries were investigated in girls with IS and matched controls, in which early skeletal overgrowth with catch-down growth affecting the right, but not left, upper arm was observed. However, due to limited specimen availability, the iliac cartilage could only be obtained for analysis from the donor site, thus bilateral histomorphometric comparisons could not be made on this iliac crest cartilage. In the present study, histomorphometric analysis of the iliac crest cartilage revealed significantly thicker hypertrophic zones and a larger area of cell-nest in the hypertrophic zone, and significantly increased mean number of cells in the cell-nests in both the proliferative and hypertrophic zones in IS patients compared with those in control subjects with Risser 0 and Oxford stage 2–3. These findings indicate that in the early stage of pubertal growth, the growth activity of IS patients was significantly higher than that of maturity-matched controls. However, in patients with Risser grade 2, there was no observable difference in terms of the above parameters between IS patients and their controls. These findings support our hypothesis that there is an accelerated systemic longitudinal growth in IS patients that is only active during the early stage, but growth is normal at the late stage, of puberty when compared with that of controls. This phenomenon supported by data from several previous reporting anthropometric measurements of scoliotic girls that show they are taller than controls in early puberty, even without correcting for the height loss caused by scoliosis, but these difference disappear after maturity [5, 8, 14]. Similarly, in a study conducted by Burwell *et al*. [40], skeletal sizes-for-age as standard deviation scores (SDSs) for limb segment lengths were compared between right-handed girls with right thoracic AIS (RT-AIS) and normal girls. They found that early systemic skeletal overgrowth is associated with significant corrections in three of four upper limb segments, but not in lower limb segments. The peripheral catch-down to skeletal overgrowth is also consistent with our results.

Several limitations of the present study should be considered. First, we acknowledge the marked limitations of these preliminary results. Limited patient data were available to increase the sample population size, and no comparisons could be made with other results because of the absence of previous studies. Second, the IS patients had a different gender distribution than the controls, though it was not significant. Because IS is seen predominantly in girls, we assumed the gender distribution difference would be inevitable. Differences in pubertal growth by gender have been well-recognized, as males have different growth activities compared with females of the same chronological age during puberty. Although the Oxford grading was also used in this investigation to support the Risser grading, it must still be recognized that these indicators are not very precise. With these concerns, all the subjects enrolled in this study were well-matched by skeletal maturity as assessed by the Risser grade and Oxford stage, in combination with YSM, instead of the chronological age. Third, the present study did not include any specimens from Risser grade 1, 3 or 4 because of the small number of patients in our cohort with Risser grade 1 (two IS patients and no controls) and the impossibility of obtaining iliac cartilage from the iliac crest in patients with Risser grades 3 and 4. Fourth, many factors such as a patient’s genetic make-up, gender, the Risser grade, and chronological age can influence the curve type, severity, and flexibility index. Endochondral activity has not been analyzed by scoliosis curve type, severity (Cobb angle), or flexibility index. The severity and rigidity of the curve may depend mainly on the length of the disease history, growth activity, and strength of the para-spinal connective tissues, among other reasons. The endochondral activity shows the risk of curve progression. Therefore, the endochondral activity has not been analyzed by scoliosis curve type, severity (Cobb angle), or flexibility index. Finally, iliac cartilage was chosen to represent the systemic endochondral ossification activity in the present study, and endochondral ossification of the controls was assumed to represent the endochondral ossification of normal healthy adolescents. It is unreasonable to assume that the growth activity of iliac cartilage is the same as other areas, and there may be differences between the controls in the present study and other normal, healthy adolescents. In a study conducted by Nicolopoulos *et al*. [41], an increased pelvic height for age was found in girls with IS, which may indicate that the iliac cartilage growth plates are for age developmentally advanced of the growth plates in other skeletal regions. However, as the typical source of autograft bone for fusion during surgery, the iliac crest cartilage was the only source free from mechanical loading we could obtain. Furthermore, we assume it is ethically impossible to obtain growth plates from the other skeletal regions and from normal healthy controls.

Because the modulation of endochondral bone formation, like long bones, is controlled by both local and systemic factors, further studies should focus on matrix synthesis, as well as local and systemic factors to understand the underlying mechanisms that cause the differences observed here.

## Conclusions

A histomorphometric comparison of the endochondral growth activity in IS and skeletal maturity matched control patients was performed. An accelerated endochondral growth was shown in IS patients in early pubertal stage (Risser grade 0 with Oxford grade 2–3), but not in late pubertal stage (Risser grade 2). These findings indicated an accelerated endochondral growth in IS patients during the early stage of puberty.

## Authors’ original submitted files for images

Below are the links to the authors’ original submitted files for images. Authors’ original file for figure 1 Authors’ original file for figure 2 Authors’ original file for figure 3
