# Supplementary figures and images for: Accelerated endochondral growth in adolescents with idiopathic scoliosis: a preliminary histomorphometric study
Source: BMC Musculoskelet Disord. 2014 Dec 13;15:429. doi: 10.1186/1471-2474-15-429 (PMC4301996; doi:10.1186/1471-2474-15-429)

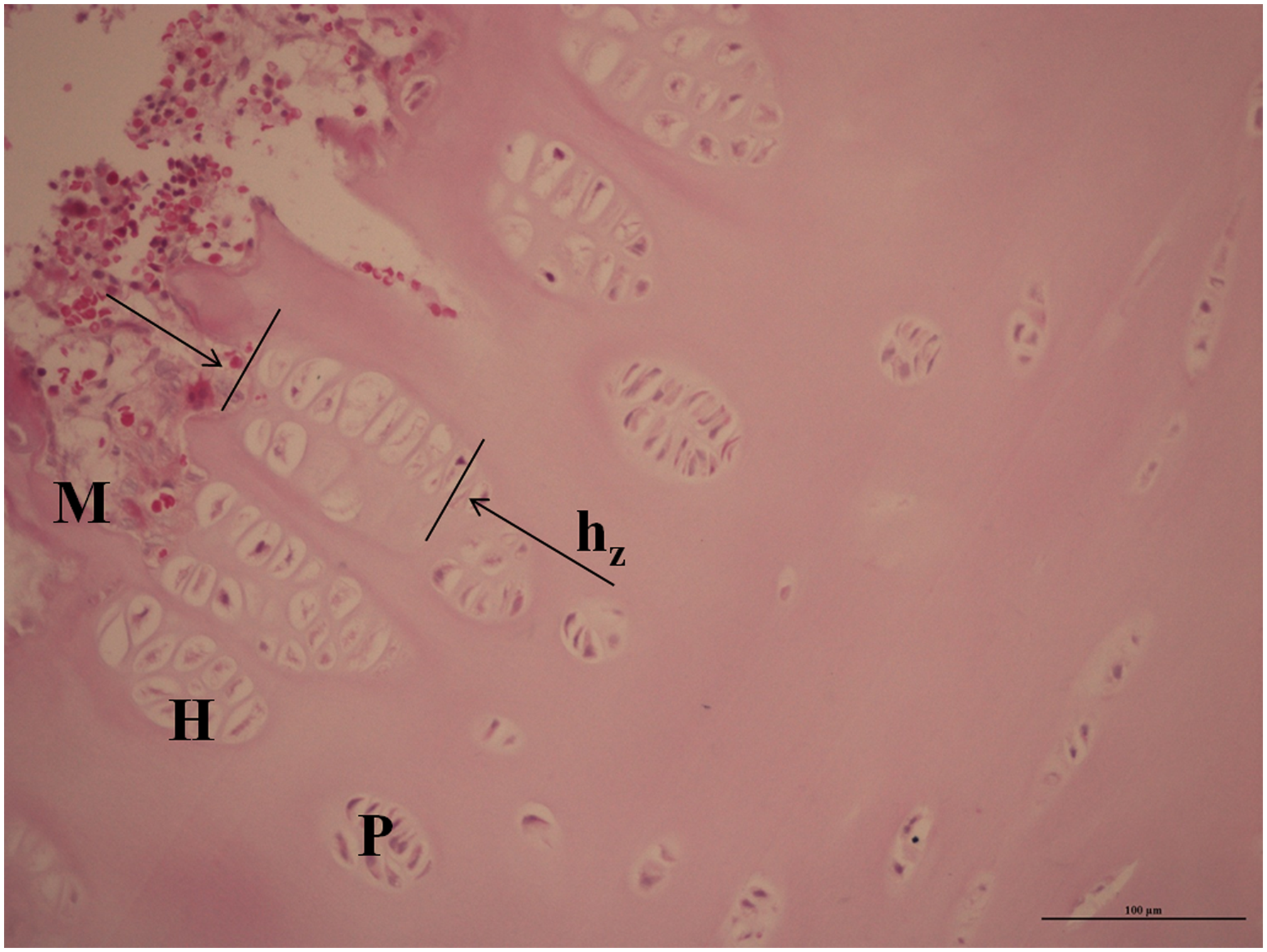

Supplement: Supplementary file 1 — Authors’ original file for figure 1 [file 12891_2013_2388_MOESM1_ESM.tif]

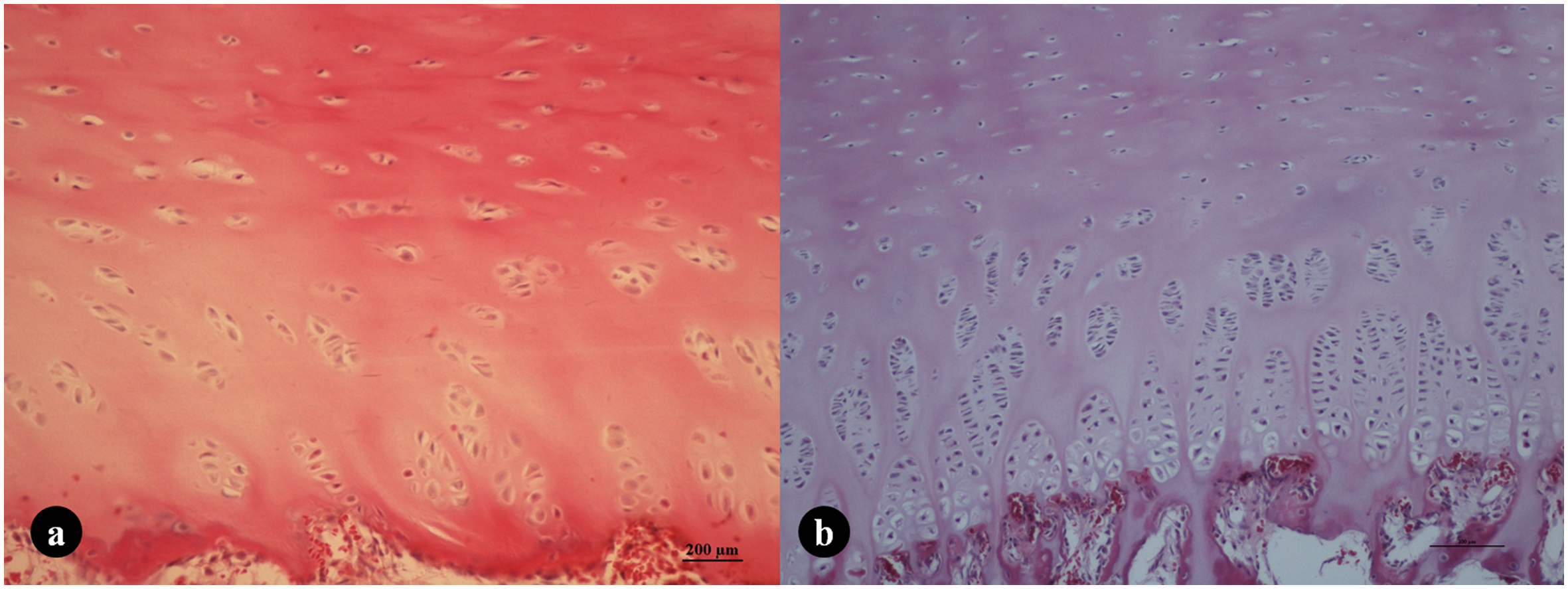

Supplement: Supplementary file 2 — Authors’ original file for figure 2 [file 12891_2013_2388_MOESM2_ESM.tif]

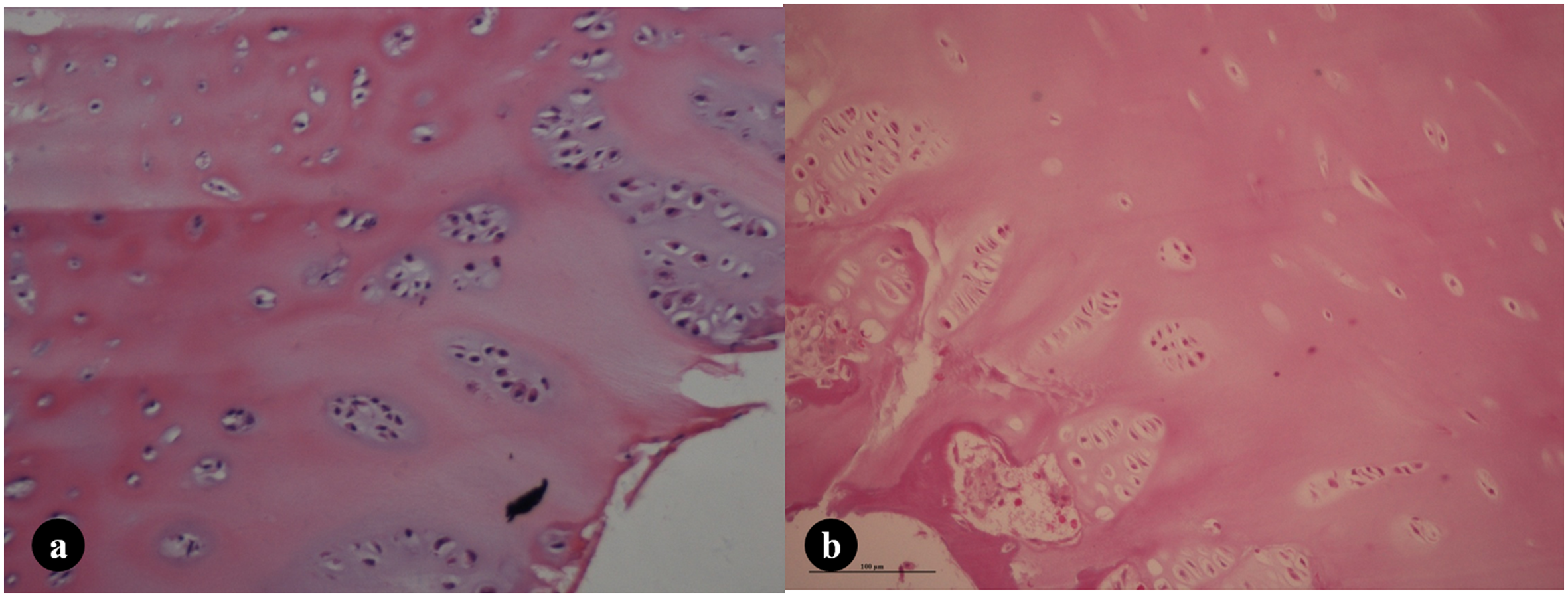

Supplement: Supplementary file 3 — Authors’ original file for figure 3 [file 12891_2013_2388_MOESM3_ESM.tif]
